# Gene signatures and prognostic values of m6A regulators in clear cell renal cell carcinoma – a retrospective study using TCGA database

**DOI:** 10.18632/aging.101856

**Published:** 2019-03-15

**Authors:** Jingcheng Zhou, Jiangyi Wang, Baoan Hong, Kaifang Ma, Haibiao Xie, Lei Li, Kenan Zhang, Bowen Zhou, Lin Cai, Kan Gong

**Affiliations:** 1 Department of Urology, Peking University First Hospital, Beijing, P.R. China; 2 Institute of Urology, Peking University, Beijing, P.R. China; 3 National Urological Cancer Center, Beijing, P.R. China; 4 Department of Urology, Fudan University Shanghai Cancer Center, Shanghai, P.R. China; 5 Department of Oncology, Shanghai Medical College, Fudan University, Shanghai, P.R. China.; * Equal contribution

**Keywords:** N6-Methyladenosine, m6A, renal cell carcinoma, prognosis

## Abstract

m6A is the most common form of mRNA modification. However, little is known about its role in clear cell renal cell carcinoma (ccRCC). This study aims to identify gene signatures and prognostic values of m6A regulators in ccRCC. In this study, a total of 528 ccRCC patients from TCGA database with sequencing and CNV data were included. Survival analysis was performed using log-rank tests and Cox regression model. The association between alteration of m6A regulators and clinicopathological characteristics was examined using chi-square test. The results showed that alteration of m6A regulators was associated with pathologic stage. Patients with any CNVs of the regulatory genes had worse OS and DFS than those with diploid genes. Moreover, deletion of m6A “writer” genes was an independent risk factor for OS, and copy number gain of “eraser” genes could magnify the effect in a synergistic way. Additionally, low expression of the writer gene *METTL3* was related to activations of adipogenesis and mTOR pathways. Thus, we for the first time determined genetic alterations of m6A regulators in ccRCC and found a significant relationship between the alterations and worse clinical characteristics. The findings provide us clues to understand epigenetic modification of RNA in ccRCC.

## Introduction

Renal cell carcinoma (RCC) is the most lethal cancer in genitourinary system. The latest cancer statistic report shown that more than 65,000 new cases were diagnosed in the U.S. resulting in almost 15,000 deaths every year, which made it the sixth most common tumor site for male [1]. Clear cell renal cell carcinoma (ccRCC) is the most common type (~80%) of renal cell carcinomas. Clinically, up to 16% of patients with ccRCC are metastatic at the time of diagnosis, and the 5-year relative survival rate is only 12%. Although developments in medical oncology and surgery have revolutionized the approach to ccRCC, the prognosis has only marginally improved. As to localized ccRCC, 20% to 30% of patients experience recurrence after primary treatment and currently no approved therapies are found to reduce the risk of recurrence, progression or death [2,3]. In recent years, while target therapy has been proven to prolong survival in metastatic patients, the median survival is still less than 3 years [4]. Furthermore, drug resistance and financial burden are considerable problems in clinical practice [5]. Thus, exploring the molecular mechanism underlying the pathogenesis of ccRCC and new therapeutic targets are still challenging issues.

The genetic and epigenetic alterations of DNA and histone have been widely studied in tumor progression and led to the development of many therapeutic modalities including histone deacetylase inhibitors and drugs targeting hypoxia related pathway [6,7]. Apart from the above two molecules, the role of RNAs in diverse cellular processes attracted extensive attention and became a fast growing field in the last decade. More than one hundred of chemically modified nucleotides are found in different types of RNAs, including rRNA, tRNA, mRNA, snRNA and others [8]. The modified RNAs, especially mRNA, play critical roles in the post-transcriptional regulation of gene expression. In eukaryotes, m6A is the most common form of mRNA modification, the abundance of which has been found to be 0.1–0.4% of total adenosine residues [9–11]. m6A is proved to be widespread throughout the transcriptome, and actually it is present in the mRNAs of over 7,600 genes and in more than 300 noncoding RNAs [12]. In general, m6A is highly conserved between human and mouse, located in 3’UTRs, around stop codons and the long internal exons [13], leading to alterations of RNA stability, splicing, intracellular distribution and translation [14–17]. The cellular m6A status is mediated by a group of genes called “writers” (WTAP, METTL3 and METTL14), “erasers” (FTO and ALKBH5) and “readers” (YTHDF1, YTHDF2, YTHDF3, YTHDC1 and YTHDC2) [18]. The writers form a multisubunit methyltransferase enzyme complex and upregulate the m6A level, while erasers are m6A demethylase enzymes, which make the event reversible. Furthermore, the readers are effectors that decode the m6A methylation information and transform it into a functional signal.

m6A dysregulation is involved in diverse cellular process and causes decreased cell proliferation, impaired self-renewal capacity, developmental defects and cell death [19]. It has been reported that the alterations of m6A regulatory genes play an important role in the pathogenesis of a variety of human disease including obesity, impairment of spermatogenesis, neuronal disorders and immunological disease. More recently, the alterations of m6A regulatory genes have been shown to promote progression of both breast cancer and hematologic malignancies through cancer stem cell formation and abnormal differentiation state maintenance [20,21]. Another study also proves that METTL3, a major RNA N6-adenosine methyltransferase, promotes liver cancer progression through YTHDF2 dependent post-transcriptional silencing of SOCS2 [22]. While m6A is found to be associated with tumorigenesis in different types of cancers, little is known about the relationship between m6A-related genes and ccRCC. Hence, in this study, we analyze the clinical and sequencing data of ccRCC cohort from TCGA, and evaluate the alteration spectrum of ten m6A regulatory genes in ccRCC as well as the association between the genetic alterations and clinicopathological characteristics including survival.

## RESULTS

### Mutations and CNVs of m6A regulatory genes in ccRCC patients

**Figure 1:**
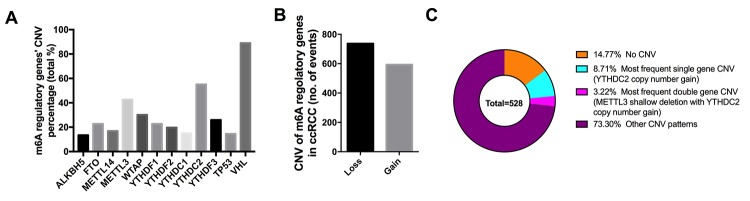
**CNVs of m6A regulatory genes in ccRCC.** (**A**) Percentage of ccRCC samples with CNVs of the m6A regulators based on the data from TCGA. (**B**) Events of copy number gain or loss of m6A regulatory genes in ccRCC samples. (**C**) The most common patterns of CNVs in m6A regulatory genes in ccRCC samples.

Among the 451 cases with sequencing data, mutations of m6A regulatory genes were found merely in 19 independent samples (Table 1); however, CNVs of the ten m6A regulatory genes were frequently observed in 528 ccRCC samples with CNV data (Figure 1A). In detail, the m6A “reader” gene *YTHDC2* had the highest frequency of CNV events (55.11%, 291/528) followed by *METTL3* (30.11%, 159/528), which are am6A “reader” and “writer” gene. Furthermore, we also observed frequent CNVs of *VHL* (89.02%) and *TP53* (14.58%) in this cohort in line with published literatures [23].

**Table 1:** Mutations of m6A regulatory genes in 451 ccRCC patients.

**ccRCC Sample ID**	**ALKBH5**	**FTO**	**METTL14**	**METTL3**		**WTAP**	**YTHDF1**	**YTHDF2**	**YTHDC1**	**YTHDC2**
TCGA-B0-5698										S598T
TCGA-CJ-6033								D130N		
TCGA-A3-3346							V519Cfs*2			
TCGA-A3-3374								D80*,S151R		
TCGA-A3-3382										V871I
TCGA-AS-3778										X499_splice
TCGA-A3-3362										E775V
TCGA-CJ-4636				X506_splice, G486S,E481K						
TCGA-B0-5098						T91A				
TCGA-B0-5099									Y31C	
TCGA-BP-4801						M163I				
TCGA-BP-5199		S256R								
TCGA-CJ-4905									E483*	
TCGA-CZ-4853			S351C							
TCGA-CZ-5459				D499H						
TCGA-B0-5695							F425S			
TCGA-CJ-5678										L804*
TCGA-CJ-5682									N97H	
TCGA-CW-5581										D987Y

Next, we evaluated the CNV patterns in ccRCC samples and found that most of the CNV events led to loss of copy number (737/1331) (Figure 1B, Table 2), which was similar as the CNV status in AML [24]. Copy number gain of *YTHDC2* was the most frequent alteration in all the CNVs of m6A regulatory genes (Figure 1C), and the simultaneous shallow deletions of *METTL3* and *YTHDC2* also ranked first among the concurrence of CNVs in two genes, implying the importance of m6A writer genes in the process of RNA m6A modification.

**Table 2:** Different CNV patterns occur in ccRCC samples (n=528).

		**Diploid**	**Deep deletion**	**Shallow deletion**	**Copy number gain**	**Amplification**	**CNV sum**	**Percentage**
Eraser	**ALKBH5**	457	0	45	26	0	71	13.45%
	**FTO**	408	0	22	98	0	120	22.73%
Writer	**METTL14**	439	3	74	11	1	89	16.86%
	**METTL3**	303	0	207	18	0	225	42.61%
	**WTAP**	369	1	150	8	0	159	30.11%
Reader	**YTHDF1**	408	0	0	119	1	120	22.73%
	**YTHDF2**	424	0	95	9	0	104	19.70%
	**YTHDC1**	448	0	65	14	1	80	15.15%
	**YTHDC2**	237	1	7	230	53	291	55.11%
	**YTHDF3**	391	1	72	61	3	137	25.95%
	**TP53**	451	2	50	25	0	77	14.58%
	**VHL**	58	58	405	7	0	470	89.02%

### Alterations of m6A regulatory genes were associated with clinicopathological and molecular characteristics

Then, we assessed the relationship between alterations (CNV and/or mutation) of m6A regulatory genes and the clinicopathological characteristics of patients. The results revealed that alterations of m6A regulatory genes were significantly associated with higher Fuhrman Nuclear Grade (Table 3). Due to the fact that *VHL* and *TP53* play important roles in the pathogenesis of ccRCC [25], we further evaluated if the variation of m6A regulatory genes was related to the alterations of these two genes. As expected, the alterations of m6A regulatory genes were significantly correlated with *VHL* and *TP53* alteration; in fact, only 1 sample was absent from alterations of m6A regulatory genes among the 57 patients with *TP53* alteration (Table 4).

**Table 3:** Clinical pathological parameters of ccRCC patients with or without mutation/CNV of m6A regulatory genes.

		With mutation and/or CNV*	Without mutation and CNV*	**P**
**Age**	<=60	32	218	0.325
	>60	25	225	
**Gender**	Female	26	148	0.069
	Male	31	295	
**Pathological Stage**	I	36	206	0.123
	II	7	48	
	III	8	113	
	IV	6	73	
	Discrepancy	0	3	
**Historical Grade**	G1	5	6	**<0.001**
	G2	33	175	
	G3	14	182	
	G4	4	74	
	x	1	4	
	N/A	0	2	
**T stage**	T1	36	212	0.104
	T2	8	57	
	T3	12	164	
	T4	1	10	
**N stage**	N0	25	201	0.683
	N1	1	17	
	Nx	31	225	
**M stage**	M0	51	353	0.133
	M1	6	71	
	Mx	0	19	

*With mutation or CNV: Cases have mutant or CNV or mutant+CNV, confirmed through TCGA database. Without mutant and CNV: Cases with neither mutant nor CNV, confirmed through TCGA database. Ambiguous variables (Nx, Mx, N/A, discrepancy and Gx) were excluded from chi-square test or non-parametric test

**Table 4:** Relationship between molecular characteristics and m6A regulatory genes alteration in ccRCC patients.

		Without mutation or CNV*	With mutation and CNV*	**χ^2^**	**P**
**VHL**	wt	8	25	4.362	**0.037**
n=495	alteration	49	413		
**TP53**	wt	56	316	12.041	**0.001**
n=456	alteration	1	83		

**Figure 2:**
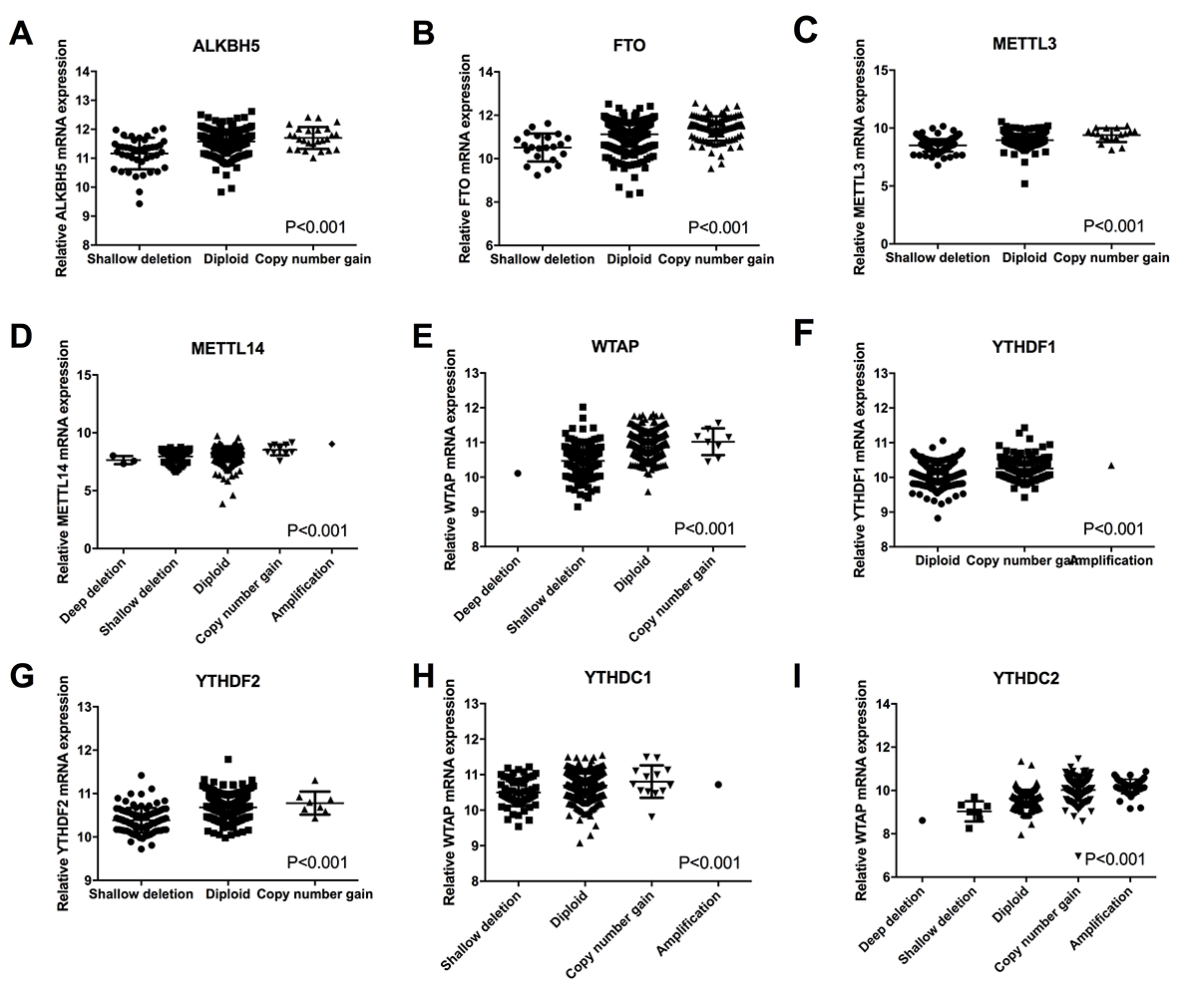
Correlation between different CNV patterns and mRNA expression levels of ten m6A regulatory genes respectively.

The effects of alterations in m6A regulatory genes on the mRNA expression were next evaluated. The results showed that the mRNA expression levels were significantly associated with the diverse CNV patterns in 525 ccRCC samples. For all the ten genes, copy number gains were related to higher mRNA expression; while, deep deletions or shallow deletions resulted in a decline of mRNA expression (Figure 2). Furthermore, we have done some immunohistochemistry staining for METTL3 and METTL14 protein in tissue arrays containing about 130 pairs of ccRCC tissue and normal tissue to confirm the findings. The results were in line with our analysis, which showed METTL3 and METTL14 were highly expressed in ccRCC tissues than normal (P<0.05) (Figure S1). However, due to the lack of prognosis data, we couldn’t perform the survival analysis in this study.

### Association between CNVs of m6A regulatory genes and survival of ccRCC patients

**Figure 3:**
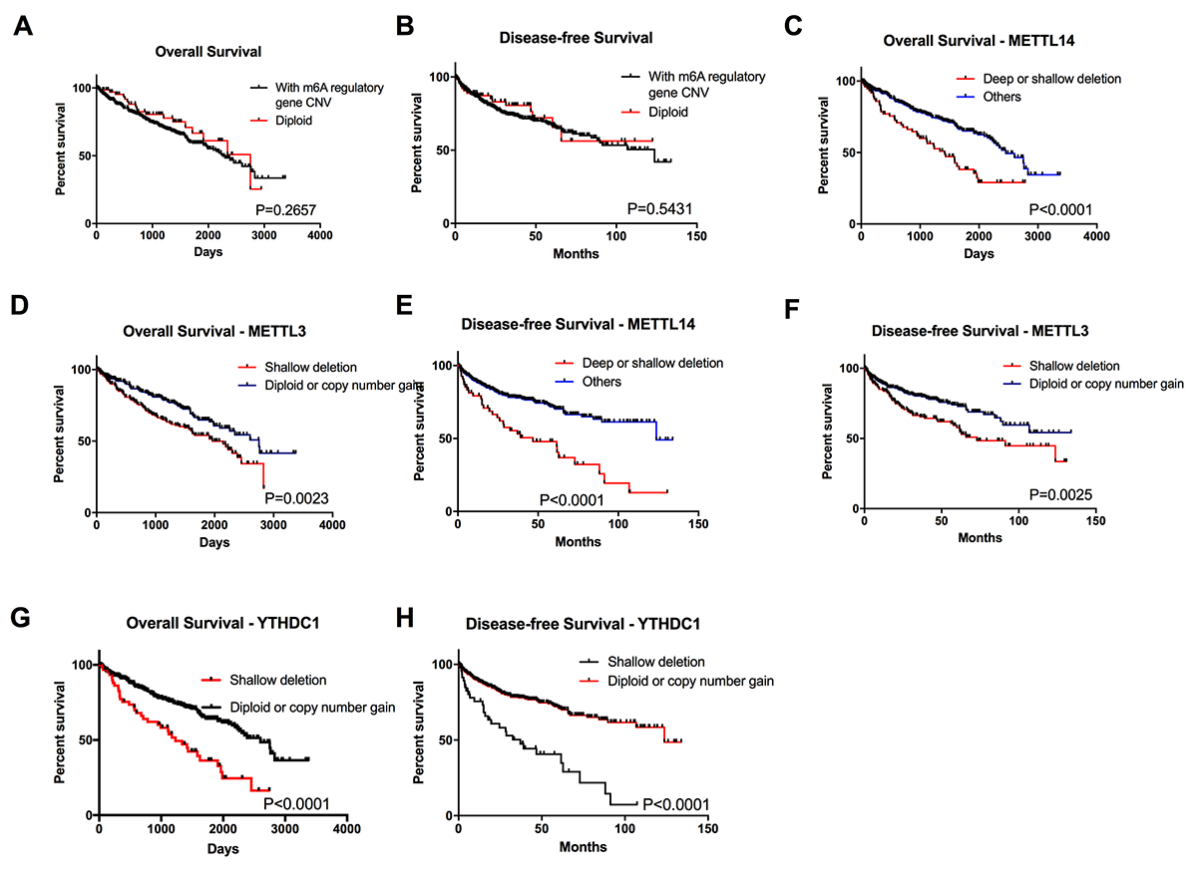
**Overall survival of ccRCC patients with CNVs of m6A regulatory genes.** (**A-B**) OS and DFS of patients with any CNVs of m6A regulatory genes or with diploid genes. (**C-H**) OS and DFS for patients with different CNV types of *METTL3, METTL14* and *YTHDC1*.

To explore the prognostic value of CNVs in the m6A regulatory genes, we analyzed the effects of CNVs on the overall survival (OS) and disease-free survival (DFS) among ccRCC patients, and found that individuals with or without m6A regulatory genes’ CNVs didn’t have any correlation with OS and DFS (Figure 3A-B). Furthermore, separate analysis of the ten genes revealed that patients affected by deletions of *YTHDC1, METTL14 or METTL3* (one reader and two writer genes of m6A) had poorer OS and DFS (Figure 3C-H); while, no significant difference was observed between different subgroups based on the CNVs of the other ten m6A regulatory genes (Figure S2). Multivariate Cox regression analyses demonstrated that alteration of m6A regulator genes was an independent risk factor for overall survival (Table 5). Considering the writer genes are a group of methyltransferase enzymes and the most important part in m6A regulation process, the results implied that the down-regulation of m6A level might be associated with poor patient survival.

**Table 5:** Univariate and Multivariate COX regression analysis of m6A regulatory genes for ccRCC patients' overall survival (OS) and disease-free survival (DFS)*.

	**OS**					**DFS**			
**Variable**	**Univariate**		**Multivariate**			**Univariate**		**Multivariate**	
	HR (95% CI)	P	HR	P		HR (95% CI)	P	HR	P
Age (>60 vs <=60)	1.787(1.294-2.467)	**0.000**	1.714(1.096-2.68)	**0.018**		1.419(0.997-2.018)	0.052		
Gender (male vs female)	1.078(0.782-1.485)	0.648				1.398(0.945-2.069)	0.094		
Stage (I-II vs III-IV)	4.370(3.116-6.128)	**0.000**	1.313(0.54-3.192)	0.548		6.426(4.327-9.545)	**0.000**	4.499(1.92-10.541)	**0.001**
M (M1 vs M1)	4.400(3.185-6.080)	**0.000**	2.701(1.594-4.575)	**0.000**		7.876(5.411-11.463)	**0.000**	3.133(1.819-5.394)	**0.000**
N (N1 vs N0)	2.880(1.524-5.442)	**0.001**	1.468(0.735-2.933)	0.277		3.851(1.888-7.855)	**0.000**	2.078(1.01-4.276)	**0.047**
T (T3-T4 vs T1-T2)	3.645(2.643-5.028)	**0.000**	1.608(0.719-3.596)	0.247		4.413(3.063-6.359)	**0.000**	0.734(0.354-1.526)	0.408
Grade (3-5 vs 1-2)	2.842(1.972-4.098)	**0.000**	2.037(1.205-3.442)	**0.008**		3.337(2.21-5.039)	**0.000**	1.577(0.926-2.688)	0.094
TP53 (altered vs diploid)	1.300(0.853-1.980)	0.222				1.193(0.732-1.946)	0.479		
VHL (altered vs diploid)	0.945(0.555-1.610)	0.835				1.130(0.608-2.103)	0.699		
m6A regulator alteration (Writer loss + Eraser gain vs others)	1.495(1.043-2.142)	**0.028**	1.69(1.006-2.84)	**0.047**		1.654(1.102-2.485)	**0.015**	1.463(0.819-2.614)	0.199

*Ambiguous variables (Nx, Mx, N/A, discrepancy and Gx) were excluded.

**Figure 4:**
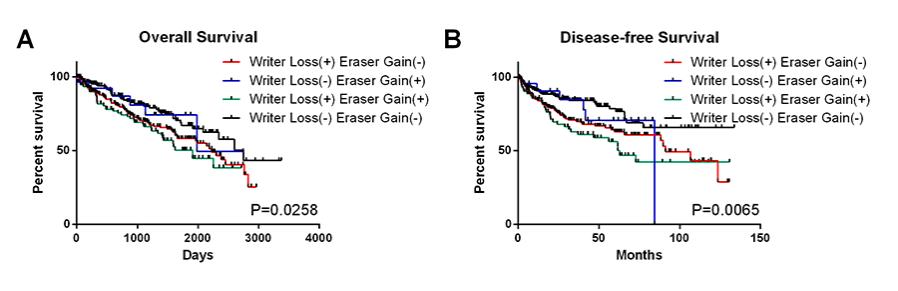
OS and DFS of ccRCC patients with simultaneous alterations of writer genes and eraser genes.

In order to confirm the above conclusion, we next tested it among patients who were affected by two kinds of CNVs (deletions of writer genes and copy number gain of eraser genes). As the result showed, patients with deletions of writer genes in combination with copy number gain of eraser genes had worse OS and DFS than those with only deletions of writer genes (Figure 4A-B). This provided more evidence for the relationship between down-regulated m6A level and poor survival.

### Enrichment analysis of *METTL3* loss of function

**Figure 5:**
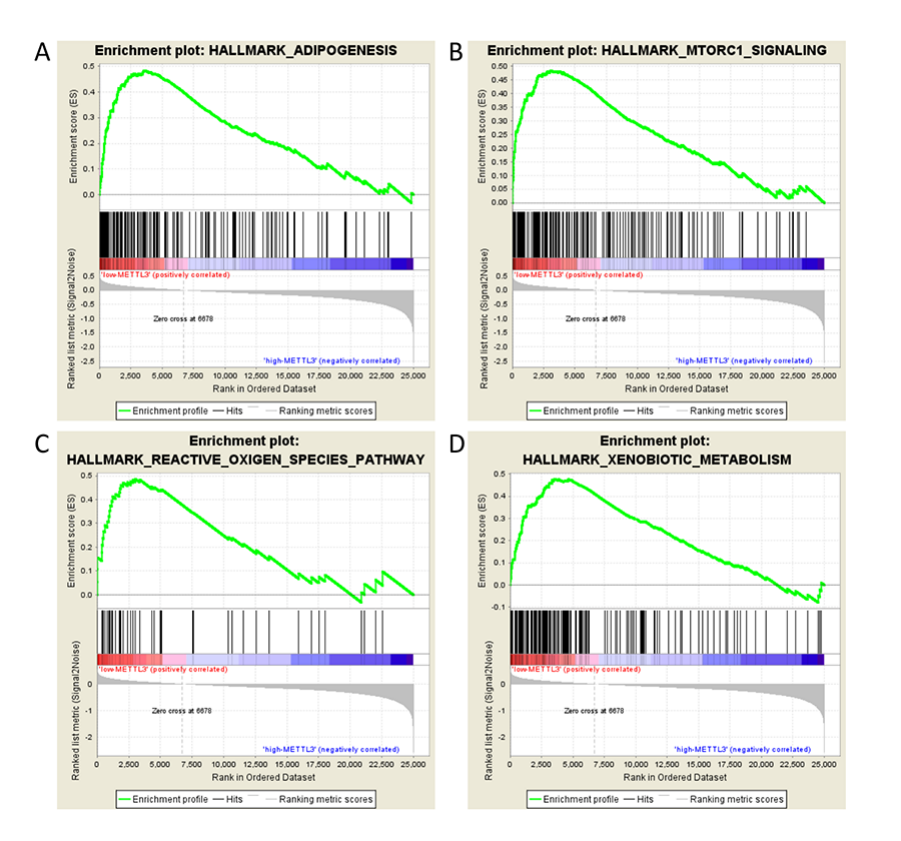
**GSEA results of different expression level of *METTL3*.** Gene set enrichment plots of (**A**) adipogenesis, (**B**) mTORC1 signaling, (**C**) reactive oxygen species, and (**D**) xenobiotic metabolism pathways related to low *METTL3* mRNA level in the ccRCC samples.

Given the importance of METTL3 in the methylation process and the interesting results we found, we determined to explore the role of m6A dysregulation in the pathogenesis of ccRCC. We examine the enriched gene sets in samples with different *METTL3* mRNA expression levels. The GSEA analysis suggested that low METTL3 expression was associated with some critical biological processes including adipogenesis, mTOR pathway and reactive oxygen species (ROS) (Table 6 and Figure 5), giving a clue of the underlying mechanism in the pathogenesis of ccRCC. To validate our findings, we examined the expressions of genes related to the pathways above. We found that several genes of adipogenesis and mTORC1 signaling pathways were upregulated in RCC tissues (Figure S2H), which partially validate the GSEA results. Besides, several studies have found that METTL3 could participate in adipogenesis and mTORC1 signaling pathways, which are consistent with our results [26,27]. Further studies are needed to illustrate the specific effect of METTL3 and METTL14 on the regulations of the downstream genes.

**Table 6:** Gene sets enrichment of low METTL3 mRNA expression level in the ccRCC cohort.

**GS DETAILS**	**SIZE**	**ES**	**NES**	**NOM *p*-val**	**FDR *q*-val**
HALLMARK_ADIPOGENESIS	190	0.48	1.72	0.028	0.227
HALLMARK_MTORC1_SIGNALING	194	0.48	1.70	0.036	0.174
HALLMARK_XENOBIOTIC_METABOLISM	194	0.48	1.61	0.027	0.232
HALLMARK_REACTIVE_OXIGEN_SPECIES_PATHWAY	44	0.49	1.57	0.048	0.228

## DISCUSSION

The transcriptome-wide mapping of m6A introduced the concept of epitranscriptome which focuses on investigating the landscapes and functions of the reversible RNA modifications in the last decade. Due to the complicated technology (m6A-Seq and m6A MeRIP) detecting the m6A level, lots of studies choose an alternative way to evaluate the genetic alterations of m6A regulatory genes and indirectly explore the association between m6A status and human diseases. Due to the tissue specificity of the “writers”, “erasers” and “readers”, the genes involved in m6A dysregulation may be different in distinct tumors. In this ccRCC cohort, the frequency of alterations of the ten m6A related genes was much higher than that reported in AML, implying that dysregulation of m6A might play a more important role in ccRCC tumorigenesis compared with AML [24]. Furthermore, there was a high frequency of concurrent alterations of two regulatory genes, indicating that m6A writer gene and reader gene may play synergistic roles in the process of RNA m6A modification. In addition, the “writers” *METTL3* and *METTL14* were more predisposed to mutation or CNV than the other genes in ccRCC, while alterations of the “erasers” *FTO* and *ALKBH5* were proved to be more important in breast cancer, glioblastoma and hematological malignancies [21,28,29]. The differences of involved genes between different tumor types gave us a clue that the regulation of m6A in cellular level was complicated, and future studies focusing on the m6A “writers” are needed to further clarify the regulatory mechanism of m6A in ccRCC.

Similar as CNV status in AML, most of the observed CNVs in writer genes resulted in loss of function with down-regulation of the corresponding genes, while CNVs of the eraser genes were mainly gain of function leading to up-regulation of the corresponding genes. Considering the opposite effect on m6A status for the two gene groups, these alterations eventually decreased the m6A level in ccRCC. In accordance to our results, many studies on other solid tumors, like breast cancer and glioblastoma, also observed the down regulated m6A level [21,30]. This may be explained by the connection between m6A and differentiation pathways that control cancer stem cell fate [31]. The activation of hypoxic pathway was a hallmarker in tumorigenesis of the above three solid tumors. A study showed that hypoxia was related to increased breast cancer stem cell formation directly through upregulating ALKBH5 or indirectly through ZNF217/METTL3-METTL14-complex pathway. The consequent decreased level of NANOG mRNA m6A modification resulted in the upregulation of NANOG expression, which was a key transcription factor that is associated with pluripotency. Interestingly, overexpression of NANOG was also observed in ccRCC tissue compared with normal tissue [32]. Considering the high frequency of inactivation of *von Hippel-Lindau* (*VHL*) gene and the upregulated hypoxia induced factor-α (HIF- α) in ccRCC, we hypothesize that there may exist a m6A regulatory pathway (VHL-HIF-ZNF217-METTL3/METTL14) in ccRCC cells, leading to the formation and maintenance of ccRCC cancer stem cells. More work is needed to be done to verify the pathway.

We also evaluated the effect of m6A regulatory gene alterations on survival of ccRCC patients. In line with the characteristics of genetic alterations of m6A related genes, the writer genes *METTL3* and *METTL14* are the only two genes among the ten regulators that are associated with the overall and disease-free survival, which reconfirms that the writers were main regulators of m6A in ccRCC. Although the effect of METTL3 became insignificant in the multivariate Cox regression model, we should take it into consideration that METTL3 and METTL14 need to form a complex to function as RNA methyltransferases, and thus the two molecules are related to each other, possibly resulting in the statistically insignificance. A worst overall survival in patients with writer gene loss of function in combination with eraser gene gain of function was observed, making it clear that decreased level of m6A plays a significant role in ccRCC progression. However, we failed to reach any significant results referred to interactions between the combined genetic alteration and DFS, possibly because of the limited number of patients with writer gene loss of function in combination with eraser gene gain of function. Direct detecting the m6A level and evaluating its effect on ccRCC survival in a new cohort is needed to illustrate this contradictory phenomenon.

Series of cancer related pathways are dysregulated in ccRCC development. We found in this ccRCC cohort that low METTL3 mRNA expression level was associated with activated adipogenesis and mTOR pathway, which are two very important cellular processes in ccRCC development. Similar as our results, a recent study showed knockdown of *METTL3* in ccRCC cell lines led to obvious upregulation of PI3k, AKT and mTOR expression and patients with positive METTL3 expression had obviously longer survival time than those with negative METTL3 expression [26], implying that the mRNAs of molecules in mTOR pathway may be the target mRNA of m6A modification. Moreover, Kobayashi et al. found that WTAP-METTL3-METTL14 complex played a role in the mechanism for adipogenesis, which is consistent with our GSEA results [27]. In addition, we observed the alteration of m6A regulatory genes was significantly associated with *VHL* mutation and *TP53* alteration. These two genes are the most important tumor suppressor genes in ccRCC. It has been reported in a human liver cancer cell line that loss of METTL3 leads to alternative splicing and gene expression changes of more than 20 genes involved in the p53 signaling pathway including *MDM2*, *MDM4*, and *P21* [13]. Also, Liu et al. found that reduced METTL3 expression could lead to reductions in m6A methylation and have an effect on AKT signaling in human endometrial cancer [33]. Thus, it is likely that genetic alterations of m6A regulators, VHL-mediated hypoxia pathway and p53-mediated cell processes act in a synergistic way to promote the pathogenesis and progress of ccRCC.

In conclusion, we for the first time determine the genetic alterations of m6A regulatory genes in ccRCC and find an obvious relationship between the alterations resulting in decreased m6A level and worse clinical characteristics including survival. It is plausible that VHL-HIF-METTL3/14 pathways are involved in the m6A regulation in ccRCC cancer cells, and PI3K-mTOR as well as p53 signaling pathways are possible downstream targets of m6A in ccRCC. To further clarify the definite target mRNA of the m6A modification during ccRCC initiation and progression, future studies in another ccRCC cohort with m6A-Seq and m6A MeRIP will be helpful to confirm our findings.

## MATERIALS AND METHODS

### Ethics statement

All of these clinical data, CNV, mutation, mRNA expression data were retrieved from TCGA program by cBioportal platform [34] and TCGA-assembler [35] which are open to the public under some guidelines. So it is confirmed that all written informed consent was achieved.

### Data processing

Within the TCGA database, we identified 528 ccRCC patients with CNV data and pathology reports [36]. For CNV, the loss and gain levels of copy-number changes have been identified using segmentation analysis and GISTIC algorithm. To investigate the clinicopathological significance of the status of CNV and/or mutation, this ccRCC cohort was divided into two subgroups; “with mutation and/or CNV of these ten m6A regulatory genes” and “without CNV and mutation”. The mRNA expression data were calculated from RNA-Seq V2 RSEM release, and being applied log scale before analyzing the relationship between mRNA expression and CNV.

### Gene set enrichment analysis (GSEA)

GSEA was provided by the JAVA program with MSigDB v6.1 and downloaded from the website of Broad Institute [37]. In this study, cases were divided into two groups according to the first and fourth quartile of METTL3 expression level. Finally, 18419 genes were enrolled into the GSEA process. Hallmark gene set “h.all.v6.0.symbols.gmt” was used in this study [38]. Gene sets with normalized p-value <0.05, and the false discovery rate (FDR) <0.25 were considered to be significantly enriched.

### Statistical analysis

All statistical data and figures were analyzed by using SPSS 20.0 (IBM, Chicago, USA) and GraphPad Prism 6.0 (GraphPad Software, La Jolla, CA, USA). The association between m6A regulatory genes’ CNV and clinicopathological characteristics were analyzed with chi-square test or Mann-Whitney U test. Kaplan-Meier curve and log-rank test were used to evaluate the prognosis value of m6A regulatory gene’s alteration. Cox proportional hazard regression model was performed using SPSS. All statistical results with a p-value <0.05 were considered to be significant.

## SUPPLEMENTARY MATERIAL

Supplementary Figures
