# Supplementary material for: Gene signatures and prognostic values of m6A regulators in clear cell renal cell carcinoma – a retrospective study using TCGA database
Source: Aging (Albany NY). 2019 Mar 15;11(6):1633–47. doi: 10.18632/aging.101856 (PMC6461179; doi:10.18632/aging.101856)
Supplement: Supplementary Figures [file aging-11-101856-s001.pdf]

SUPPLEMENTARY MATERIAL

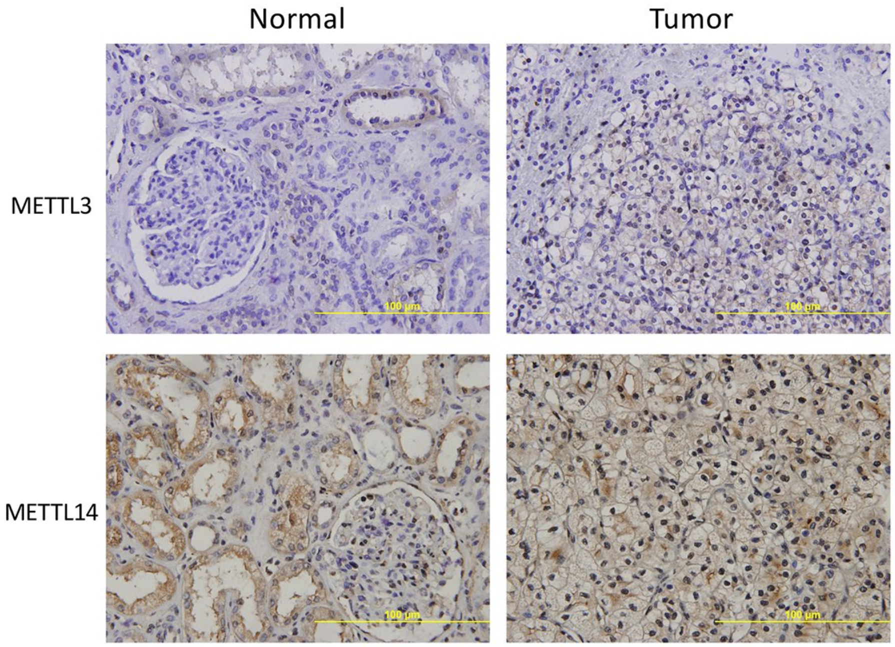

Figure S1. The protein expression of METTL3 and METTL14 in ccRCC and normal kidney tissues.

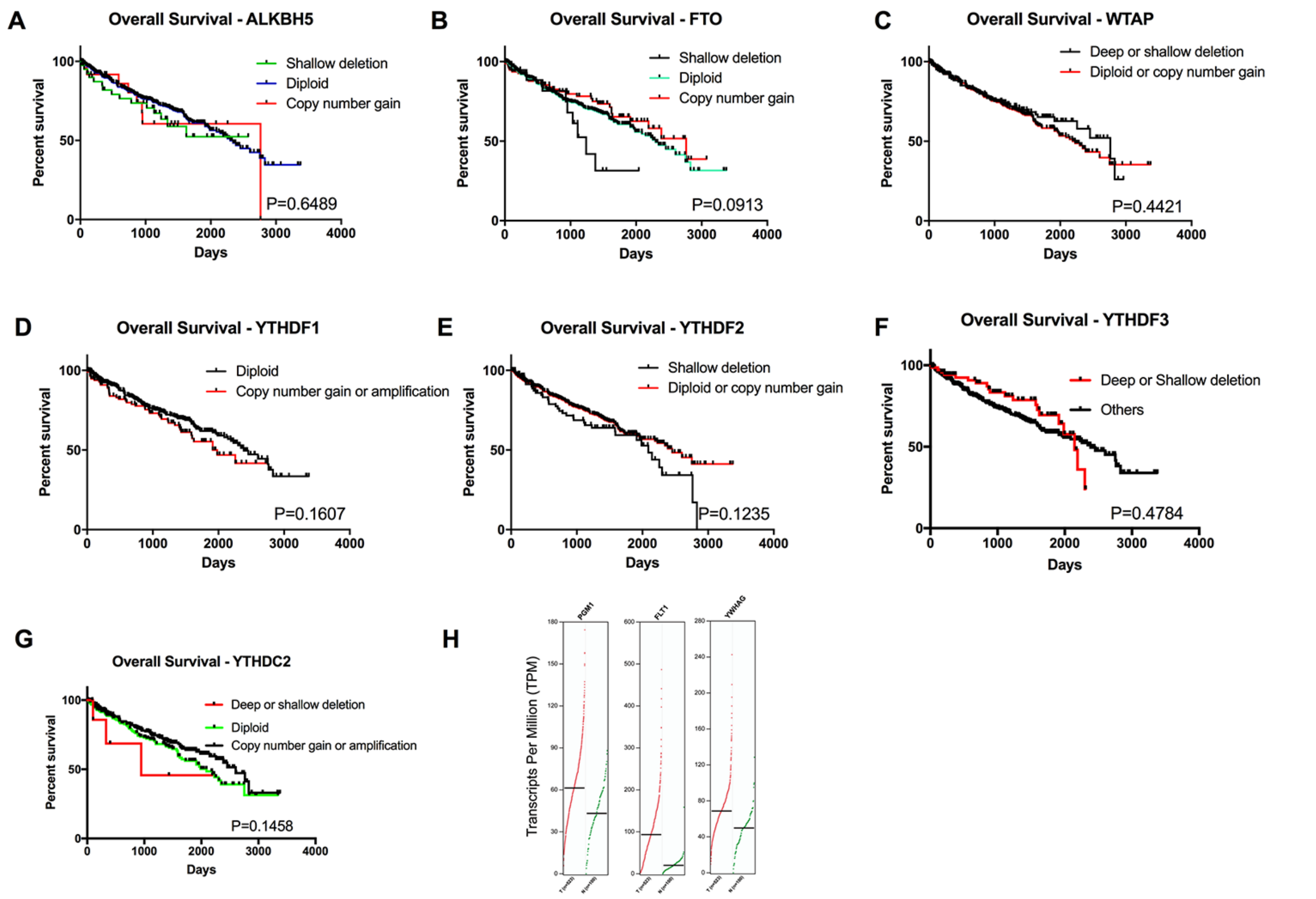

Figure S2. (**A-G**) OS of ccRCC patients with different alterations of m6A regulatory genes ALKBH5, FTO, WTAP, YTHDF1, YTHDF2, YTHDF3 and YTHDC2. (**H**) Expression of genes related to adipogenesis and mTORC1 signaling pathways in ccRCC tissues.
